# Are hospital management practices associated with enhanced quality of care for small and sick newborns? A nationwide cross-sectional study using linked inpatient admission records in Malawi

**DOI:** 10.7189/jogh.16.04054

**Published:** 2026-03-13

**Authors:** Charlotte Ward, Wanangwa Chimwaza, Vincent Samuel Phiri, Catherine Goodman, Andrew Kumitawa, Monica Malata, Alilane Linda Nyondo-Mipando, Christian Bottomley, Elias Rejoice Maynard Phiri, Samuel Ngwala, Joy E Lawn, James Cross, Eric Ohuma, Timothy Powell-Jackson, Victor Mwapasa

**Affiliations:** 1 London School of Hygiene and Tropical Medicine, London, UK; 2 Kamuzu University of Health Sciences, Blantyre, Malawi

## Abstract

**Background:**

Improved quality of care is fundamental for reducing patient mortality and building sustainable health systems. Currently, there is a lack of research on the role of hospital management in improving the quality of care and health outcomes, particularly in low-income settings.

**Methods:**

We examined associations between hospital management practices and neonatal quality of care in Malawi. We adapted the World Management Survey tool to measure 28 management practices across five domains – delivery of clinical care in the neonatal unit, human resource management, target setting, finances, and governance. In April 2022, we administered the tool to five clinical and administrative managers in each of the 36 central and first-level referral hospitals (n = 180 interviews). Further, we calculated a hospital-level management score (1 – poor, 5 – excellent) and linked these data to records of 20 831 neonatal admissions (February–July 2022). Our primary outcome was in-hospital neonatal mortality, and secondary outcomes included 14 clinical quality indicators. We examined associations between hospital-level management scores and individual-level patient outcomes using a multilevel mixed-effects Poisson regression.

**Results:**

The mean hospital-level management score across the 36 hospitals was 3.35 (standard deviation = 0.4). Among 20 831 neonatal admissions, 2590 (12.4%; 95% confidence interval (CI) = 11.9–12.8) died, representing a mortality rate of 27.2 deaths per 1000 person-days. We found no relationship between the management score and in-hospital neonatal mortality (adjusted incidence rate ratio per unit increase in the score = 1.08; 95% CI = 0.81–1.44). Five management domains were not associated with mortality, and we found limited evidence that management practice scores were positively associated with quality of clinical care.

**Conclusions:**

This study presents novel, national evidence on the association between hospital management practices and neonatal mortality in a low-income country, with complementary data on quality of clinical care. We found no evidence that hospital management practices were associated with neonatal mortality and limited evidence of an association with the quality of clinical care. Rigorous impact evaluations of targeted management interventions, with embedded process evaluations, could address potential confounders and help understand how and under what circumstances management improvements could translate into better quality of care.

A high-quality health system is a core component for achieving universal health coverage [1]. It is estimated that poor quality of care accounts for 5.7–8.4 million deaths each year in low- and middle-income countries (LMICs) [2]. Newborns represent some of the most vulnerable users of the health system, with an estimated 2.3 million neonatal deaths per year, of which a large proportion are preventable with higher quality of care [1,3]. During the Millennium Development Goals era, global and national policy was focused primarily on access, increasing the coverage of essential health services. Only recently has the quality of care risen on the policy agenda [4]. For example, a small, targeted set of interventions for newborn care has been developed as part of the Every Newborn Action Plan to reduce neonatal mortality and help reach Sustainable Development Goal (SDG) 3.2 [5]. In LMICs, there is strong evidence that health technologies, medical training, and clinical audits improve the quality of care [6]. In contrast, less research attention has been given to the broader, organisation-level factors that support health service delivery [7]. One such factor is hospital management, which is widely regarded by policymakers and the public as important but has received limited attention from empirical researchers [8,9].

Management is defined as ‘the control, monitoring or organisation of people, processes and systems in order to achieve specific goals’ [10] or ‘continuously developing the potential of an organisation to transform human and financial resources and other inputs into improved services and better health’ [11]. Because hospital management is a multidimensional concept and hospitals are complex, studies on hospital management have often relied on a case study approach within a small number of purposively selected organisations. While case studies have yielded valuable insights into management mechanisms, for example, a study of a well-performing hospital in Ghana [12], the findings may be difficult to generalise [13]. However, the potential for larger-scale studies has been constrained by the challenges in measuring and comparing management performance across facilities.

In recent years, researchers have addressed management challenges by conceptualising management as the adoption of a set of management practices, thereby focusing on the processes and systems implemented within an organisation [14]. These advances in measurement have enabled large-scale studies of the association between hospital management practices and patient outcomes. Evidence from high-income countries shows a robust link between management practices and health outcomes. Studies across eight high- and upper-middle-income countries have found that hospital management practices are associated with improved clinical outcomes, including lower all-cause mortality, fewer deaths from acute myocardial infarction, and fewer deaths from surgery [15–19]. In contrast, another study from the USA reported no association between management score and 30-day mortality [20]. In LMICs, there is limited evidence on the association between management and care processes, such as adherence to treatment, case management, and compliance with standardised protocols [21–24]. To our knowledge, there is no evidence on the association between hospital management practices and mortality in a low-income setting.

In this study, we examined whether better-managed hospitals exhibit higher adherence to quality standards for the clinical care of small and sick newborns and lower mortality rates. We developed a survey tool to systematically collect quantitative data on the management practices in Malawian hospitals. We implemented this tool in 36 tertiary and first-level referral hospitals in Malawi that provide care to >40 000 newborns per year [25]. We then linked these data to contemporaneous patient-level data on quality of clinical care practices and patient outcomes for neonates admitted to the hospital. This research was conducted as part of the Innovative Management Practices to Enhance Hospital Quality and Save Lives in Malawi (IMPRESS) project and in collaboration with the Newborn Essential Solutions and Technologies (NEST360) Alliance, an organisation partnering with governments in Malawi, Tanzania, Kenya, and Nigeria on systems strengthening package for neonatal care [26]. The project theorises that adoption of structured management practices – widely recognised as integral to a well-performing organisation – can improve the way health workers deliver care to small and sick newborns, for example, by enhancing clinical skills and knowledge, staff motivation, teamwork and communication, ultimately leading to improved quality of clinical care in the neonatal unit.

## METHODS

### Study setting and design

Malawi is a low-income country with a population of 20.9 million and a gross domestic product of USD 673 per capita in 2023 [27]. The government is the dominant provider of healthcare, alongside a well-established faith-based health sector coordinated through the Christian Health Association of Malawi (CHAM), and a small number of private not-for-profit and private-for-profit providers [28]. Through service-level agreements with the Malawi Ministry of Health, CHAM provides maternal and child health services free of charge in areas where public health facilities are not available [29]. Malawi has a district-based health system with primary care facilities and tertiary referral hospitals, as well as district hospitals serving as first-level referral centres [28]. There are four tertiary referral facilities in Malawi, known as central hospitals, which are all government-owned.

Malawi was one of the fastest-progressing countries in Africa in improving newborn survival during the Millennium Development Goals era. Malawi’s neonatal mortality rate decreased by 44% from 39 deaths per 1000 live births (range 35–45) in 2000 to 22 deaths per 1000 live births (range 19–27) in 2015 [30]. Despite past progress, neonatal mortality still remains above the SDG 3.2 target at 18.6 deaths per 1000 live births in 2022 [5,31–33]. Further, improving the quality of health services has recently become a policy priority, with the establishment of the Quality Directorate in the Ministry of Health and quality improvement teams at tertiary and district referral hospitals. Given that 97% of births in Malawi occur in health facilities [34], this investment may improve outcomes for neonates. Additionally, since 2010, several initiatives have been launched to establish best practice standards for newborn care, including the Malawi Every Newborn Action Plan and the Care of the Infant and Newborn guidelines [35]. NEST360 is a multi-partner alliance including national governments in four African countries (Malawi, Kenya, Tanzania, and Nigeria) with engineers, clinicians, and health system implementation experts [36]. Since 2018, this platform for small and sick newborns has provided life-saving technologies to neonatal units and training for service providers [26]. It also provides a health systems package, including integrated data systems for quality improvement, clinical and technical education, supervision and mentorship, and linked implementation learning materials.

We conducted an observational, descriptive cross-sectional study (survey) in 36 hospitals in Malawi, representing the largest central, district and faith-based hospitals. These hospitals have been working with the NEST360 since 2019 and include all four central government hospitals, 24 district hospitals, and the eight largest of the country’s 41 CHAM hospitals, which together account for the vast majority of secondary and tertiary care. We reported our findings according to the STROBE guidelines for observational studies [37] (Appendix S2 in the **Online Supplementary Document**).

### Procedures

Between April and May 2022, we conducted an IMPRESS Hospital Management Survey (IHMS). We adapted the World Management Survey (WMS) [15,38,39]and used it to interview hospital managers and to evaluate their responses to questions on various management practices. While our general approach was similar to that of the WMS [25], we heavily adapted the survey tool to our study context. The development and validation of the IHMS has been described elsewhere [25]. In brief, we undertook a systematic, evidence-based approach to developing the tool. First, we performed a scoping review of the quantitative tools used to measure management in a healthcare setting. Second, we reviewed relevant Government of Malawi policies and guidelines to understand formal management systems and best practices. Third, we conducted in-depth interviews with hospital managers to understand how management relates to clinical care. Finally, in a five-day workshop, we combined these findings to identify the most relevant management domains and practices for the Malawian context and developed a survey tool that was refined through iterative piloting with current and former hospital managers. The tool demonstrated strong psychometric performance in terms of reliability and validity [25].

The interviews with hospital managers covered 28 management practices across five domains: delivery of care in the neonatal unit (nine practices), human resource management for health workers (eight practices), hospital and neonatal ward level target setting and monitoring of performance (five practices), financial management (two practices), and leadership and governance (four practices) (**Table 1**). The tool contained a series of open-ended questions, designed to reveal the extent and quality of adoption of each management practice. For example, to assess whether the hospital has a standardised process for handover between staff shifts in the neonatal unit, we first asked, ‘Tell us about how handovers are done.’ We then asked ‘Do nurses regularly use written notes for handover?’ and ‘How are managers able to ensure that a standardised process for handovers is followed?’

**Table 1:** Description of management practices in the IMPRESS Hospital Management Survey and the respondents who answered questions for each management domain

Management domain and management practice	What does it test
Delivery of clinical care in the neonatal unit*	
*Layout*	How well the layout is configured to optimise patient flow.
*Triage*	If the hospital has a functioning triage system to identify, assess and provide appropriate care for newborns with life-threatening problems.
*Care of the infant and newborn protocols*	If there are standardised protocols for small and sick newborns that are applied and monitored systematically.
*Infection prevention and control protocols*	If there are standardised procedures for infection prevention and control that are applied and monitored systematically.
*Handover*	Whether the hospital has a standardised process for health workers’ handover between shifts.
*Referrals*	Whether the hospital has a standardised process for receiving neonatal referrals.
*Audit*	Whether an audit is used as an effective tool for improving clinical practices.
*Supervision*	Whether the hospital has a system in place for supervising health workers in the neonatal unit.
*Equipment*	Whether the hospital has a system in place for preventive maintenance and repair of equipment in the neonatal unit.
Human resource management for health workers†	
*Appraisal*	Whether the hospital has a formal system to appraise the performance of healthcare workers.
*Promotion*	Whether the promotion of health workers is based primarily on job performance.
*Reward*	Whether good individual performance is rewarded (financial or otherwise) proportionately.
*Poor performance*	Whether the hospital can deal with underperformers (including the use of staff sanctions).
*Recruitment*	Whether the hospital has the ability to identify and recruit skilled health workers on a permanent basis.
*Temporary staff*	Whether a hospital can forecast and address gaps in critical staff through temporary and locum workers.
*Staff allocation*	Whether the hospital allocates health workers to roles they are best qualified for.
Hospital and neonatal ward level target setting and monitoring of performance†	
*Capacity strengthening*	Whether the hospital has a programme for capacity strengthening to improve the skills of health workers.
*Monitoring errors*	Whether the hospital has systems in place for detecting harmful practices.
*Performance review*	Whether hospital managers monitor hospital performance of patients and quality of care indicators in the hospital.
*User satisfaction*	Whether the hospital uses patient or family feedback and uses evidence for improvement.
*Target range*	Whether the targets for the hospital and neonatal unit cover a sufficiently broad set of metrics.
*Target communication*	Whether targets are easily understandable and openly communicated.
Financial management‡	
*Budget setting*	Whether the hospital has a consultative and systematic process for setting the annual budget.
*Budget expenditure*	Whether the hospital has an up-to-date statement of hospital revenue and expenditure.
Leadership and governance†	
*Senior leadership governance*	Whether the hospital has a functional hospital management team.
*Quality of care governance*	Whether the hospital has a functional quality improvement support team and a neonatal ward-level work improvement team.
*Drug procurement*	The functionality of the procurement systems to get medicines and supplies for the neonatal unit.
*Infection prevention and control governance*	Whether the hospital has a functional IPC programme.

IPC – infection prevention and control

*Respondents included the sister-in-charge of the neonatal unit and the unit matron.

†All five respondents were included.

‡Respondents included administrators, district nursing officers, and district medical officers.

To evaluate hospital managers’ responses, the tool included a scoring grid on a 1–5 scale. The interviewers were guided in scoring by detailed descriptions of management practices typically present in a hospital, with scores of one, three, and five. For staff handovers, for example, one was defined as ‘there are no systems in place for health workers to pass information between each other between their shifts,’ whereas a score of five was defined as ‘a standardised process exists for handover and is used by clinicians and nurses. The process is regularly monitored for compliance.’ If interviewers felt they were unable to make an accurate assessment based on the tool’s questions, they were trained to probe further by asking for examples until they were confident in their scoring of the practice. We also included two alternative approaches to scoring management practices in the IHMS. The first, a simpler version of our main approach, was based on a closed-ended question (which preceded the open-ended questions) for each management practice. For example, for staff handovers the interviewee was asked ‘Do you have a standardised process for health workers’ shift handover? If yes, how often do staff comply with the standardised process?’ The second, a record review, was based on actual observation of the presence or absence of management-related items, such as meeting minutes, clinical manuals, administrative documentation, and forms. For staff handovers, research assistants recorded the presence or absence of a handover book in the neonatal unit (Appendix S1 in the **Online Supplementary Document**). Our sister paper, which tested correlations among hospital management measures, found that the primary management measure was highly correlated with the two alternative scoring methods [25].

The interview teams included research assistants with postgraduate health-related qualifications and hospital work experience. We provided one week of intense training and continued to supervise the teams during data collection. Given that respondents in certain roles may be better informed about some management practices, we interviewed five different types of manager per hospital to reduce measurement error and limit the influence of any single respondent. The five respondents – sister in charge of the neonatal unit, unit matron, hospital administrator, district nursing officer, and district medical officer – answered questions from management domains that are most appropriate for their role (**Table 1**). Research assistants worked in pairs as the interviewer and the scorer. During the three-day visit to each hospital, the interview teams also completed the record review. The data were collected on tablets using Open Data Kit (University of Washington, Seattle, Washington, USA).

We used patient-level data from an electronic health records system, the Neonatal Inpatient Dataset, as described in detail elsewhere [36]. This comprised a deidentified prospective dataset collecting data on outcomes and care pathways for every newborn admitted to a newborn care unit in NEST360-implementing hospitals. The dataset included the newborn’s admission diagnosis, microbiology and laboratory results, interventions received and survival status at discharge. Each hospital has a dedicated data entry clerk who uses primary-source documentation, such as patient forms or clinical case notes (*e.g.* Neonatal Admission Form and Critical Care Pathway Form), to enter data into a structured REDCap form (Vanderbilt University, Nashville, Tennessee, USA). The system has automated and human data quality checks, and the data server is hosted by the Ministry of Health in Malawi.

### Hospital management measures

To generate our primary measure of hospital management, for each management practice, we computed the mean of respondents’ scores within each hospital and calculated the mean across all 28 management practices. We refer to this measure as the IHMS score, ranging from 1–5. The aggregation method implies that each management practice contributes equally to the overall score, and we chose this approach because *a priori*, we did not know which management practices were likely to be more or less correlated with performance. This is also the approach used by the WMS. We followed the same method to generate management scores in each domain.

We calculated scores using two alternative approaches to measuring management. Using closed-ended questions, we coded responses on a 0–1 scale and calculated the mean across the 28 management practices. Further, using the record review, we coded the responses on a 0–1 scale and computed the mean across the 25 items. We interpreted both alternative measures as proportions of the maximum score obtainable. To facilitate comparisons of results across management measures, we computed Z-scores by subtracting the mean and dividing by the standard deviation (SD).

### Outcomes

The primary outcome was the in-hospital mortality of babies admitted to the neonatal unit. Furthermore, we examined 14 secondary outcomes that assessed the quality of clinical care. These were rigorously defined through consultations with clinicians in Malawi and neonatal experts, based on the World Health Organization (WHO) guidelines [40] and adapted to the Malawian context using the Care of Infant and Newborn guidelines [41]. Some indicators, such as measuring oxygen saturation on admission, should be measured and recorded for all neonates. Others are condition-specific, such as administering antibiotics to neonates with suspected clinical sepsis; hence, the denominator varies across indicators, reflecting the number of neonates who should have received each specific intervention (Table S1 in the **Online Supplementary Document**).

### Other variables

Patient characteristics included weight at admission, sex, inborn status (*i.e.* whether the baby was born in the hospital), length of stay, calendar month of admission, and reason for admission. The latter was a categorical variable indicating the primary reason for admission to the neonatal unit as stated by the health worker in the patient records. We derived facility characteristics from two data sources. The hospital type (district government or CHAM) and the hospital’s geographical zone were obtained from the Neonatal Inpatient Dataset. The other characteristics were derived from quality improvement visits for small and sick newborn care conducted quarterly by the Malawian government with support from NEST360. For this analysis, we used data for quarters one to three in 2022. These data included the number of beds in the neonatal unit (the sum of cots, radiant warmers, and incubators), number of babies on the neonatal unit on the day of the quality improvement (QI) visit, neonatal bed occupancy (the number of babies on the neonatal unit on the day of the QI visit/neonatal unit capacity), number of health workers (doctor, nurse, clinical officer) on the neonatal unit on the day of the QI visit or on the night before the QI visit.

### Statistical analysis

We conducted the analysis at the individual patient level. We linked the patient data to hospital management scores and other hospital variables via hospital identifiers. Our patient sample comprised infants admitted to the neonatal units between 1 February 2022 and 31 July 2022, to align with the timing of the management survey. We excluded infants with an admission weight of 1000 g or less because the study hospitals were not sufficiently resourced to provide specialised care for such vulnerable infants, thereby limiting any role for management in influencing outcomes. We imputed the missing data for the admission weight variable using the birthweight variable, and in the absence of birthweight, we used the mean admission weight. For all other variables, missing data were handled by exclusion. We compared missingness between hospitals with low and high management scores to assess whether there were systematic differences between these groups (Table S2 in the **Online Supplementary Document**).

We described patients’ and facilities’ characteristics across the entire analytical sample and disaggregated them by hospitals with IHMS scores above and below the median. We examined the association between each management score and mortality using a Poisson’s regression, with hospital included as a random effect to account for clustering within hospitals, and length of hospital stay included as an offset (*i.e.* we modelled the rate of death per person day in hospital). We fitted three models: an unadjusted model, an adjusted model that included patient characteristics to account for differences in case-mix between hospitals, and an adjusted model that included both patient and hospital characteristics. We explored associations using different measures of management.

The secondary outcomes measured the uptake of clinical interventions, and we analysed these using the same statistical approach. However, we did not include length of stay as an offset, as these outcomes reflect actions that should be taken regardless of how long the patient is in hospital. One set of regressions replaced the overall IHMS score with a domain-specific score called the delivery of clinical care in the neonatal unit. We hypothesised that management practices in this domain are more likely to influence the uptake of clinical interventions than those in other management domains.

We performed several sensitivity analyses for the primary outcome: inclusion of a data collection ‘team’ variable, exclusion of patients with missing data on admission weight, use of a birthweight variable (instead of admission weight), inclusion of babies under 1000 g, inclusion of variables related to the mother’s health status and type of delivery, weighting each hospital equally, and adjusting for clinical staff (Table S3 in the **Online Supplementary Document**).

We also performed sensitivity analyses for the primary outcome, where we adjusted the analysis period. To explore reverse causality, we conducted a sensitivity analysis on the association between IHMS score and mortality in the 12 months after the IHMS survey, controlling for the baseline mortality estimate (six months prior to the IHMS survey) (Table S4 in the **Online Supplementary Document**). We performed analyses using Stata, version 18.5 (StataCorp LLC, College Station, Texas, USA) and RStudio, version 2023.12.1 + 402 (Posit, Boston, Massachusetts, USA).

## RESULTS

### Descriptive results

**Figure 1:**
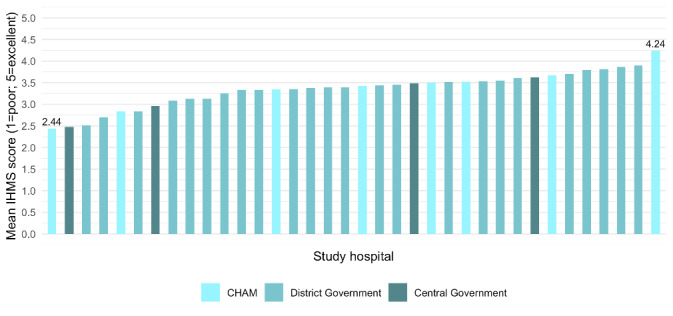
Management practice scores across Malawian hospitals, by facility type. CHAM – Christian Health Association of Malawi, IHMS – IMPRESS Hospital Management Survey.

We included 36 hospitals with complete data on management practices. The mean IHMS score was 3.35 (SD = 0.41), ranging from 2.87 (SD = 0.59) for the target setting and monitoring of performance domain, to 3.59 (SD = 0.46) for the delivery of clinical care in the neonatal unit domain. The mean IHMS score was  3.13 (SD = 0.52) for central hospitals, 3.38 (SD = 0.35) for district, and 3.37 (SD = 0.54) for CHAM hospitals. Mean IHMS scores ranged from 2.44 to 4.24 (**Figure 1**; Figure S1 in the **Online Supplementary Document**).

Capacity in terms of the absolute number of neonatal unit beds (cots, radiant warmers, and incubators) was greater in hospitals with above median management scores (mean (x̄) = 36.6, SD = 23.7) compared to those with below median management scores (x̄ = 30.7, SD = 15.7). The occupancy rate was lower in hospitals with management scores above the median (x̄ = 0.76, SD = 0.2) than in those with scores below the median (x̄ = 0.95, SD = 0.3).

A total of 21 370 neonates were admitted to the neonatal units of the study hospitals between 1 February and 31 July 2022. Of these, 539 (2.5%) were excluded because their weight was ≤1000 g. Among the remaining 20 831 neonates, 2590 (12.4%) died. The mortality rate was 27.2 deaths per 1000-person-days of admission across the entire sample. The mortality rate was 25.6 deaths per 1000-person-days of admission in those admitted to hospitals with above median management scores, whereas it was 28.6 deaths per 1000 person-days in those admitted to hospitals with below median management scores. Further, 52.7% of deaths occurred within 48 hours of admission to the neonatal unit. The primary causes of death were neonatal hypoxic ischemic encephalopathy (25.0%), respiratory distress syndrome or prematurity (13.8%), and sepsis (suspected or culture positive) (10.8%) (Table S5 in the **Online Supplementary Document**). The proportion of female admissions was 45.1% in hospitals with management scores above the median, compared with 43.7% in hospitals with scores below the median. The mean length of stay in the neonatal unit was 5.1 days in hospitals with above median management scores and 4.2 days in hospitals with below median management scores. All other patient characteristics were comparable between hospitals with above or below median management scores (**Table 2**). Adherence to clinical practice was comparable between hospitals with above and below median management scores for the majority of indicators (Table S1 in the **Online Supplementary Document**). A scatter plot of the mean IHMS and neonatal mortality for each of the 36 hospitals is presented in Figure S2 in the **Online Supplementary Document**.

**Table 2:** Descriptive statistics about patient and hospital characteristics*

Variable	Worse management†	Better‡	Total§
Hospital management practices			
*IHMS score, x̄ (SD)*	3.05 (0.3)	3.66 (0.2)	3.35 (0.4)
Neonatal outcome			
*Dead*	1472 (12.1)	1118 (13.0)	2590 (12.4)
Total person days of admission to the neonatal unit	51 533	43 656	95 189
Mortality rate per 1000 person-days of admission	28.6	25.6	27.2
Sex			
*Female*	5342 (43.7)	3884 (45.1)	9226 (44.3)
Inborn status¶			
*Outborn*	2720 (22.3)	1971 (22.9)	4691 (22.5)
Admission weight in g, x̄ (SD)	2610.2 (749.2)	2544.9 (749.0)	2583.2 (749.8)
Length of stay in neonatal unit in days, x̄ (SD)	4.2 (8.1)	5.1 (9.0)	4.6 (8.5)
Type of hospital			
*Central government*	2 (11)	2 (11)	4 (11)
*District government*	13 (72)	11 (61)	24 (67)
*CHAM*	3 (17)	5 (28)	8 (22)
Geographical zone			
*Central Eastern*	2 (11)	3 (17)	5 (14)
*Central Western*	1 (6)	6 (33)	7 (19)
*Northern*	2 (11)	5 (28)	7 (19)
*South Eastern*	6 (33)	2 (11)	8 (22)
*South Western*	7 (39)	2 (11)	9 (25)
Neonatal unit capacity, x̄ (SD)║	23.4 (12.3)	24.4 (18.5)	23.9 (15.5)
Number of babies on the neonatal unit on the day of QI visit, x̄ (SD)	12.6 (9.8)	9.8 (10.2)	11.2 (10.0)
Neonatal unit occupancy, x̄ (SD)**	0.8 (0.4)	0.7 (0.3)	0.7 (0.3)

CHAM – Christian Health Association of Malawi, IHMS – IMPRESS Hospital Management Survey, QI – quality improvement

*Presented as n (%) unless specified otherwise.

†Hospitals with management score below the median. N = 12 211 admissions in 18 hospitals.

‡Hospitals with management scores above the median. N = 8 620 admissions in 18 hospitals.

§N = 20 831 admissions in 36 hospitals.

¶Inborn refers to a baby born in the same hospital where she/he is admitted to the neonatal unit.

║Number of cots, radiant warmers, and incubators per hospital, for Q1–3 in 2022.

**Number of babies on the neonatal unit on the day of QI visit/neonatal unit capacity, for Q1–3 in 2022.

### Association with mortality

The incidence rate ratio (IRR) of 1.08 (95% confidence interval (CI) = 0.81–1.44) indicates that for every one-unit increase in management score, neonatal mortality increases by 8.0% (**Table 3**). However, this increase was not statistically significant. The lower confidence interval limit implies that, at best, for every one-unit increase in management score, neonatal mortality was reduced by 19.0%. There is also no evidence that any domain-specific management measures or alternative management measures are associated with mortality. Moreover, the results were very similar across the three alternative measures of management, as shown by the point estimates of the Z-scores.

**Table 3:** Association between hospital management scores and neonatal mortality

Items	Model 1		Model 2*		Model 3*	
	**IRR (95% CI)**	***P*-value**	**IRR (95% CI)**	***P*-value**	**IRR (95% CI)**	***P*-value**
IHMS score (scale 1–5)	1.25 (1.01–1.54)	0.038	1.21 (0.99–1.48)	0.067	1.08 (0.81–1.44)	0.592
Domain of management (1–5 scale)						
*Delivery of clinical care*	1.12 (0.88–1.43)	1.359	1.13 (0.90–1.41)	0.296	1.08 (0.86–1.36)	0.514
*Human resources*	1.23 (1.03–1.47)	0.020	1.21 (1.03–1.43)	0.019	1.12 (0.87–1.44)	0.400
*Target setting and monitoring*	1.13 (0.981.32)	0.098	1.09 (0.94–1.27)	0.266	0.97 (0.81–1.16)	0.739
*Financial management*	1.19 (1.00–1.41)	0.047	1.18 (0.98–1.42)	0.074	1.09 (0.92–1.28)	0.316
*Leadership and governance*	1.13 (0.86–1.48)	0.374	1.05 (0.79–1.38)	0.750	0.86 (0.66–1.12)	0.273
Measure of management (Z-score)						
*IHMS score*	1.10 (1.00–1.19)	0.038	1.08 (0.99–1.17)	0.067	1.03 (0.92–1.16)	0.592
*Close-ended survey score*	1.09 (1.02–1.17)	0.012	1.09 (1.01–1.16)	0.018	1.04 (0.90–1.19)	0.590
*Record review survey score*	1.07 (0.99–1.17)	0.101	1.06 (0.98–1.14)	0.149	0.99 (0.90–1.10)	0.919

CI – confidence interval, IHMS – IMPRESS Hospital Management Survey, IRR – incidence rate ratio

*Model 1 is unadjusted. Model 2 controls for patient factors (admission weight, sex inborn status, month, admission reason). Model 3 controls for patient and facility factors (neonatal unit occupancy, neonatal unit capacity, hospital type, geographical zone).

### Association with quality of care

**Figure 2:**
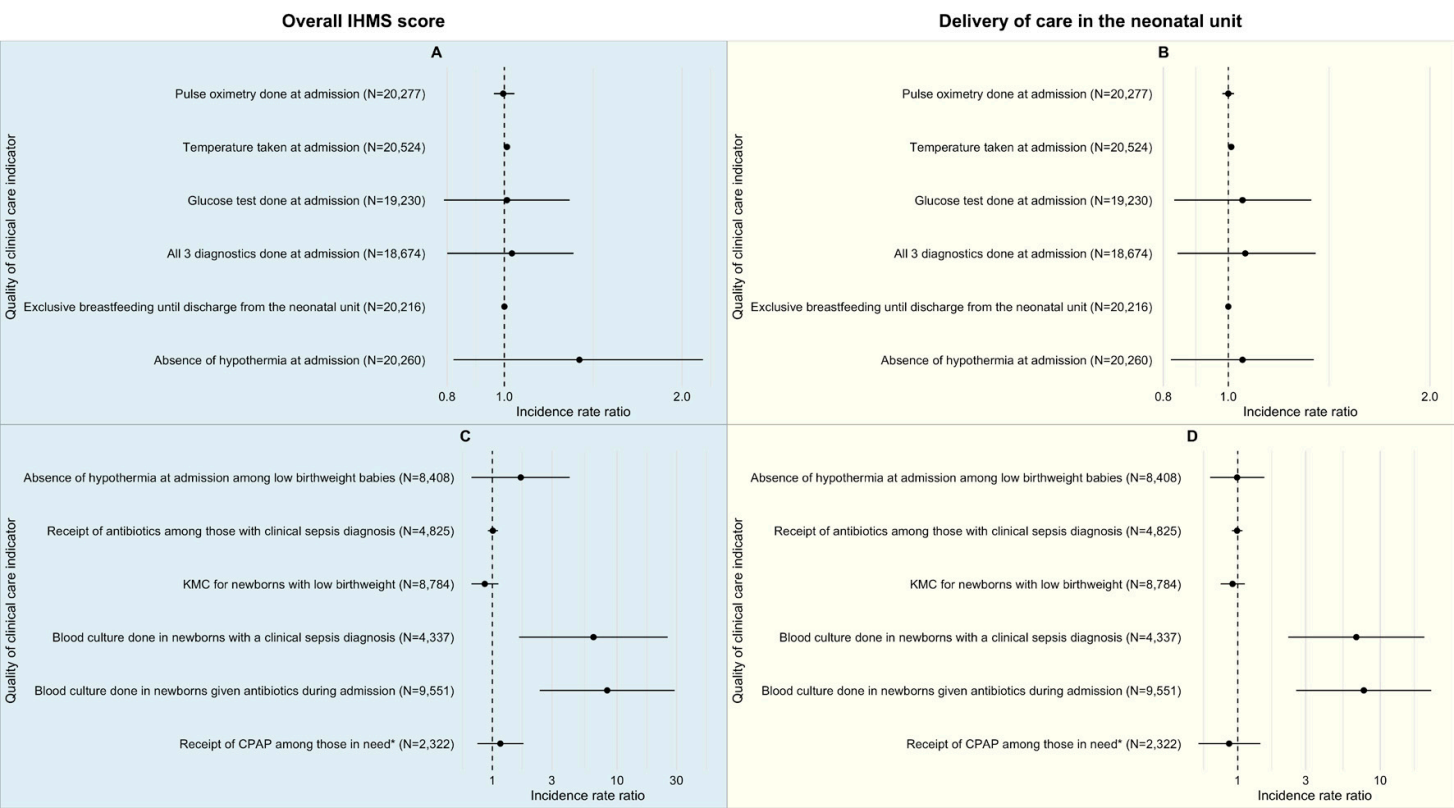
Association between overall IHMS score and quality of clinical care. The figure reports incidence rate ratios for a one-unit increase in IHMS score from a Poisson’s regression model, with 95% confidence intervals. The model uses patient-level controls (admission weight, sex, inborn status, month, admission reason) and facility-level controls (neonatal unit occupancy, neonatal unit capacity, hospital type, geographical zone). **Panel A.** Overall IHMS score. **Panel B.** Domain-specific IHMS score. **Panel C.** Overall IHMS score. **Panel D.** Domain-specific IHMS score. Indicators in Panels A–B measure adherence to interventions that should be administered to all neonates admitted to the neonatal unit. The indicators in Panels C–D measure condition-specific interventions that should be administered to a subset of neonates. The results for the indicator ‘bilirubin testing for those admitted with jaundice’ and ‘phototherapy done for jaundice’ were omitted from Panels C–D because of the small sample size and sparse data. *Those in need include two groups of babies: with birthweight 1000–1499 g, and with birthweight 1500–1999g and respiratory distress syndrome and hypoxia. CPAP – continuous positive airway pressure, KMC – kangaroo mother care, N – number of neonates eligible for the clinical intervention.

We compared IHMS scores with quality-of-care indicators for interventions that should be provided to all admitted neonates. For five out of six indicators, there was no evidence of an association between IHMS score and quality of care after controlling for patient and facility characteristics. The results were qualitatively similar when we replaced the overall score with the domain-specific management score for delivery of clinical care in the neonatal unit (**Figure 2**, Panels A and B). Further, there was strong evidence for an association between better managed hospitals and adherence to blood culture for those with a clinical sepsis diagnosis (IRR = 6.5; 95% CI = 1.6–25.5) and adherence to blood culture for those given antibiotics during admission (IRR = 8.3; 95% CI = 2.4–28.9). When we replaced the overall score with the domain-specific management score for clinical care delivery in the neonatal unit, the results were qualitatively similar (**Figure 2**, Panels C and D). The full results for all the statistical models are available in Table S6 in the **Online Supplementary Document**. Results for the sensitivity analyses were qualitatively similar to the main analysis on the association between IHMS and neonatal mortality (Tables S3 and S4 in the **Online Supplementary Document**).

## DISCUSSION

This study presents novel, national evidence on the association between hospital management practices and neonatal mortality in a low-income country, with complementary data on the quality of clinical care. We found no evidence of an association between management practices and neonatal mortality, a result that held across management domains and alternative measures of management. Evidence from other studies on the association between management and hospital mortality is mixed [42], with some evidence that better-managed hospitals are associated with reduced mortality [15–19], whereas others report no association [20,43–45]. Studies to date analysing the association between management and mortality have only been conducted in high-income, upper-middle-income, or lower-middle-income countries (USA, UK, Sweden, Germany, Canada, Italy, France, Brazil, China, and India).

We found limited evidence of an association between better hospital management (both overall and in the domain ‘Delivery of care in the neonatal unit’) and the quality of clinical care. Although better-managed hospitals showed higher adherence to blood culture protocols, only a minority of facilities have the laboratory capacity to perform blood cultures [46]. Since all hospitals are included in the denominator regardless of laboratory capacity, this association is driven by a small subset of equipped facilities and should be interpreted cautiously. Furthermore, many indicators showed high levels of adherence across hospitals, reducing variation and the potential to detect associations with management practices. Among indicators being performed across all hospitals and with room for improvement, we observe no significant association with management practices. These findings contribute to a mixed body of evidence. For example, two studies conducted in the USA and UK hospitals found a positive association between a WMS score and process-of-care measures for acute myocardial infarction and other clinical conditions [16,47]. In LMICs, however, the evidence is more variable. A study in Tanzanian health facilities reported that higher management scores were associated with correct treatment and compliance with infection prevention and control measures [24]. Similarly, research in Kenyan facilities found that adherence to structured human resource practices, such as written job descriptions and regular supervision, was associated with higher-quality care during clinical consultations [22]. However, a cross-sectional study across five African countries found no significant association between management practice score and the quality of voluntary medical male circumcision [21].

A strength of this study is that we measure the association between hospital management and health outcomes, with complementary data on clinical quality to give a more complete picture of how management could lead to improvements in patient health. Another strength is that, our work demonstrates the feasibility of linking management data with clinical outcomes at scale in a low-income setting.

The results of this study should be interpreted in light of its limitations. First, as an observational study, we cannot infer causality. In particular, there may be unmeasured confounding factors affecting the association between management and mortality. For example, although we adjusted for patient case mix, residual confounding may persist if better-managed hospitals serve disproportionately sicker populations. Reverse causality is also a potential concern, whereby good managers are purposefully sent to hospitals based on their performance, although we note that this allocation could be to both well-performing and poorly-performing hospitals and therefore is unlikely to create systematic bias. A sensitivity analysis (Table S4 in the **Online Supplementary Document**) confirms that reverse causality is unlikely. Second, we were limited to an effective sample size of 36 hospitals, which could have reduced the power of the analysis and contributed to the null findings. Third, our study relied on clinical data, which may be subject to quality limitations, and we lack external validation. However, we found no systematic differences in missing data between hospitals, and we excluded babies weighing less than 1000 g from the analysis, as their outcomes are often not fully captured in clinical records [48]. Furthermore, the Ministry of Health and NEST360 have made concerted efforts to strengthen data collection and validation processes for the neonatal inpatient dataset [49]. Fourth, we assumed that resources such as adequate staff and equipment are on the causal pathway between hospital management and quality of care, reasoning that better-managed hospitals will have more efficient systems and processes to ensure adequate drugs, equipment, and trained staff. However, there is a legitimate counterargument that if the availability of these resources is persistently low, they will undermine any meaningful impact of hospital management on quality of care and should be treated as confounders in this analysis. In practice, both perspectives could be valid for different hospitals in this sample. Our sensitivity analysis (Table S3 in the **Online Supplementary Document**) examined this, and after controlling for the number of health facility staff on the neonatal unit, the results remain qualitatively similar to those in the main analysis. Finally, experiential or perceived quality is likely to be a key driver of future utilisation of health services and adherence to discharge advice for mothers, making it an important factor in patient outcomes following discharge and a topic for future research.

There are several plausible explanations for our null findings. Here, we explore three potential explanations while acknowledging that others may also exist. First, broader resource constraints could undermine the effect of upstream organisational improvements, such as enhanced hospital management practices. For example, despite support from NEST360, assessments of hospital readiness conducted during the second, third and fourth years of the NEST360 implementation (2019–2021) in 37 Malawian hospitals identified persistent gaps in medical equipment, laboratory capacity, pharmacy supplies, and biomedical support, with readiness scores ranging between 33–53% [50]. While improvements may have occurred by 2022, gaps are likely to remain. Another critical constraint is the availability of human resources for health, which has been identified as a strategic priority by the government in the Health Sector Strategic Plan 2023–2030 [28]. An analysis conducted between 2019 and 2022 in 36 NEST360 hospitals in Malawi found that the median daytime nurse-to-baby ratio was 1:5 and the doctor-to-baby ratio was 1:105 [51]. Although no international standards exist, these figures are well below national benchmarks. For example, South Africa recommends a nurse-to-baby ratio of 1:3 in neonatal high-dependency units, and the UK recommends a ratio of 1:2 [40]. Therefore, while many of the management practices we measured could plausibly improve staff productivity, they may be insufficient in the context of chronic understaffing.

Second, management practices may have limited influence on clinical practice when the hospital lacks the autonomy to make key decisions. Despite Malawi’s formal commitment to health sector decentralisation since 1998, many crucial functions, such as hiring and drug procurement, remain centrally controlled. Hospital managers, therefore, have limited influence over functions such as hiring, promoting, and paying staff, which can reduce staff availability and retention and increase absenteeism, even in contexts of stronger management practices. Notably, the countries in which there is evidence of an association between management and mortality are in high-income settings and give hospitals considerable autonomy across most key functions [15–18,52].

Third, in resource-limited settings, management activities such as functioning quality improvement teams, performance reviews, and neonatal death audits may inadvertently divert clinical staff from patient care. All hospitals have clinician managers; however, the ability of hybrid clinical managers to maintain the more clinical aspects of their role, such as supervision, may be limited if they are not given administrative and management support to execute management practices [53]. In better-resourced contexts, such roles are often supported by dedicated administrative personnel. This additional burden in low-income settings may dilute the effectiveness of strengthened management practices.

## CONCLUSIONS

In summary, this study provides novel national evidence from a low-income country on the association between hospital management and neonatal quality of care outcomes.

We highlight that existing evidence of an association between hospital management and mortality comes from high-income, upper-middle-income, or lower-middle-income settings, in which hospitals less frequently face fundamental resource constraints. Future research should examine whether targeted management interventions, particularly when paired with structural improvements, yield measurable improvements in health outcomes. Such management interventions should be co-designed with hospital managers in situ to ensure the activities they promote are feasible and sustainable for the hospital and align with national priorities for quality improvement. Rigorous impact evaluations of targeted management interventions in settings without fundamental resource constraints will be especially valuable in addressing known and unknown confounders and assessing causality. Complimentary process evaluation data will help to unpack how and under what circumstances hospital management practices have potential to drive improvements in quality of care.

## Additional material


Online Supplementary Document
